# Prevalence and factors associated with olfactory impairment among patients with acne treated with oral isotretinoin: a cross-sectional study^☆^^☆☆^

**DOI:** 10.1016/j.bjorl.2024.101461

**Published:** 2024-06-28

**Authors:** Rogério Nabor Kondo, Hélio Amante Miot, Elouise Zwirtes Frare, Ellen Cristine Duarte Garcia, Abner Hiraku Yamakami, Marco Aurélio Fornazieri

**Affiliations:** a Universidade Estadual de Londrina (UEL), Londrina, PR, Brazil; b Universidade Estadual Paulista (Unesp), Faculdade de Medicina de Botucatu, Botucatu, SP, Brazil

**Keywords:** Acne, Anosmia, Isotretinoin, Nasal diseases, Smell disorders

## Abstract

**Objective:**

This study aimed to investigate the prevalence and factors associated with olfactory dysfunction in individuals exposed to Isotretinoin (ISO) for the treatment of acne, using the University of Pennsylvania Smell Identification Test (UPSIT®).

**Methods:**

This cross-sectional study enrolled age and sex-matched patients with acne who were current users of oral ISO and unexposed controls without olfactory complaints. UPSIT® and a validated questionnaire (Nasal Obstruction Symptom Evaluation) were administered to evaluate nasal obstruction in patients exposed to ISO.

**Results:**

A total of seventy patients were recruited, with 35 in the exposed group and 35 in the unexposed group, consisting of 18 males and 17 females in each group, aged from 17 to 47 years. The prevalence of olfactory dysfunction was higher in the exposed group compared to the non-exposed group (62.9% vs. 17.1%), yielding a Prevalence Ratio (PR) of 3.7 (95% CI 1.9–7.1). However, no participants were categorized as anosmia or severe hyposmia and the majority of dysfunction was mild hyposmia compared to moderate hyposmia (51.5% vs. 11.4%). Among the exposed individuals, gasoline, orange, coffee, and wood exhibited the highest rates of identification errors (≥54%). Olfactory function demonstrated a negative correlation with treatment duration (*p* = 0.01), cumulative dose (*p* = 0.02), and nasal obstruction (*p* = 0.02).

**Conclusions:**

Olfactory dysfunction was more prevalent among ISO users, despite the patients being unaware of the disorder. Olfactory changes were correlated with treatment duration, cumulative dose, and nasal obstruction.

**Level of evidence:**

Level 4.

## Highlights

• There was a notable prevalence of olfactory dysfunction among isotretinoin users.
• Most individuals with altered sense of smell did not self-recognize the condition.
• Olfactory changes were correlated with treatment duration and nasal obstruction.

## Introduction

Acne is the leading reason for seeking dermatological care, with an estimated global prevalence of 9.4% across all age groups.^1^, ^2^ While it is more commonly observed in adolescents, it can also affect individuals of varying ages, with approximately 98.7% of cases occurring before the age of 25.^3^ This skin condition inflicts a significant impact on quality of life and is often linked to affective disorders such as depression and anxiety.^4^

Various treatment guidelines have been proposed for acne, encompassing a spectrum from topical to systemic medications.^5^ Oral Isotretinoin (ISO) is a 13-cis-retinoic acid that has been approved by the Food and Drug Administration (FDA) since 1982 for the treatment of acne.^6^ In Brazil, it has been approved by the National Health Surveillance Agency since 1990.^7^ Although several side effects have been documented over the years, oral ISO continues to be available in the market due to its effectiveness and safety profile.

Mucosal dryness and epithelium thinning are the main adverse effects of the treatment with ISO.^8^ On the nasal cavity, dryness and epistaxis have been reported; however,^9^ limited uncontrolled studies have been conducted to assess the changes in smell function among these individuals.^10^ Despite a report of olfactory disturbance following the treatment with ISO,^11^ the medication leaflet does not list dysfunction in smell as one of its adverse effects. Meanwhile, olfactory changes can have negative impacts, as smell influences people's nutrition, safety, and overall well-being.^12^, ^13^

This study aimed to investigate the prevalence and factors associated with olfactory dysfunction in individuals exposed to ISO for the treatment of acne.

## Methods

A cross-sectional study was conducted on the population of the Municipality of Londrina, Brazil. Non-probabilistic convenience sampling was employed, involving the inclusion of all consecutive cases of acne available, provided they met the inclusion criteria and consented to participate in the study. Participants exposed to ISO and controls were matched by age and sex.

The participants aged more than 17 years without olfactory complaints, were currently users of ISO or were never exposed to it (controls). Exclusion criteria included patients with olfactory disease resulting from traumatic brain injury, those who underwent facial radiotherapy, those with reported dysfunction following airway infections (including those caused by SARS-CoV-2), and those who did not comprehend the test. Patients who were infected with COVID-19 more than a month ago and who recovered their sense of smell or who reported no olfactory loss were included in the study. The study was conducted at the Dermatologic Clinic of the University Hospital (AEHU) of the Londrina and was approved by the Research Ethics Committee of the State University of Londrina (CAAE: #48734521.5.0000.5231). All the participants signed the informed consent.

Data were collected by administering a form to patients and extracting information from the electronic medical records between July/2022, and July/2023.

The main outcome was the University of Pennsylvania Smell Identification Test (UPSIT®), which contains 40 odors, was used to assess olfactory function.^14^ This test consists of four booklets with 10 substances each, in which the individual scratches a brown stripe at the bottom of each page, smells it, and selects an option among the four alternatives. The UPSIT® score varies from 0 to 40 depending on the number of correct answers for the substances, classifying the performance of the participant as normosmia (≥32 for men and ≥35 for women), mild hyposmia (≥28 and ≤31 for men; 31≥ and ≤34 for women), moderate hyposmia (≥24 and ≤27 for men; 26 ≥ and ≤30 for women), severe hyposmia (≥17 and ≤23 for men; 19 ≥ and ≤25 for women), anosmia (≥6 and ≤16 for men; 6≥ and ≤18 for women), or a possible simulator (≥0 and ≤5 for men and women).^13^

For the participants with obstructive symptoms of the nasal airways, a validated questionnaire, the Nasal Obstruction Symptom Evaluation Scale (NOSE), was used to assess symptoms of nasal obstruction.^15^ As it was a cross-sectional study, the patients, after applying UPSIT®, were followed up on an outpatient basis to evaluate only their acne until the end of treatment with ISO.

The prevalence of olfactory dysfunction was represented by the percentiles for each substance within the groups. The effect size was estimated by the Prevalence Ratio (PR), and its 95% Confidence Interval (95% CI).

The normality of the distributions was assessed by the Shapiro-Wilk test.^16^ The comparison between quantitative variables was achieved by the Mann-Whitney test, and the comparison between qualitative variables was performed by Pearson’s Chi-Squared test, Fisher’s exact test, and Chi-Squared for trend.^17^ The correlations between the UPSIT® scores and NOSE scores with time and dose of ISO were assessed by Spearman's rank correlation coefficient (rho).^18^

All data were compiled into an Excel spreadsheet for subsequent statistical analyses. The IBM SPSS Statistics program (version 22.0) was used for statistical analysis. Significance was set as *p*-value <0.05.^19^

The sample size was estimated based on the a priori expectation of olfactory dysfunction occurring in up to 50% of the cases and 15% among the controls, with a significance level (alpha) of 0.05 and a power of 90%.^19^

## Results

A total of 70 participants (35 current users of ISO, and 35 controls) were enrolled in this study. The main characteristics of the sample are presented in Table 1, and the groups were homogeneous regarding the main baseline variables. Olfactory complaints were not reported by any participant in either group. Among the ISO group, four participants reported a history of COVID-19 infection for more than 4 months, but without olfactory complaints or impairments in the olfactory test (they presented normosmia). Smoking was more prevalent among controls, and rhinitis, among the ISO group.

**Table 1:** Main clinical and demographic characteristics of the sample.

Variables	ISO (n = 35)	Control (n = 35)	*p*-value
Age, years			
Mean (SD)	23.0 (7.3)	24.2 (6.2)	0.460
Minimum–Maximum	17−47	20−47	
Male sex, n (%)	18 (51.4)	18 (51.4)	>0.999
White race (self-reported), n (%)	25 (71.4)	24 (68.6)	0.794
Salary income per family, n (%)^a^			<0.001
≤2	2 (5.7)	23 (65.7)	
3‒4	5 (14.3)	7 (20.0)	
5‒9	22 (62.9)	2 (14.3)	
≥10	6 (17.1)	- (-)	
Schooling, n (%)			0.150
High School	16 (45.7)	22 (62.9)	
College	19 (54.3)	13 (37.1)	
Rhinitis, n (%)^b^	19 (54.3)	7 (20.0)	0.003
Current smoking, n (%)	0 (0.0)	8 (22.9)	0.005
UPSIT^c^			
Hyposmia, n (%)	22 (62.9)	6 (17.1)	<0.001
Mean (SD)	32.3 (3.0)	35.7 (2.6)	<0.001
Minimum–maximum	24–36	30–39	
NOSE^d^			
Mean ± SD	2.0 ± 2.3	–	
Minimum–Maximum	0‒12		
Treatment length^e^			
Mean (SD)	6.29 (2.4)		
Minimum–Maximum	1‒10		

a Minimum wage in Brazil in 2023.

b No flu-like symptoms at the time of the olfactory test.

c University of Pennsylvania Smell Identification Test.

d Nasal Obstruction Symptom Evaluation Scale (among those with nasal obstruction).

e In months.

All participants in the ISO group were on a daily dose of 40 mg and reported no previous relevant adverse events or interruptions in their treatment.

The olfactory function was worse among exposed individuals (UPSIT® = 32.3 vs. 35.7; *p* < 0.001). The prevalence of olfactory loss (hyposmia) was higher among individuals exposed to ISO (62.9% vs. 17.1%; *p* < 0.001), leading to a Prevalence Ratio (PR) of 3.7 (95% CI 1.9–7.1).

Regarding the severity of hyposmia, no participants were categorized as anosmia or severe hyposmia. Among the controls, there were no participants with moderate or severe hyposmia, while four (11.4%) participants in the ISO group were categorized as having moderate hyposmia. These results remained consistent when analyzed separately by gender (*p* ≤ 0.01).

There were no significant correlations between the UPSIT® scores and age, as well as education level and family income in both groups (*p* > 0.05). In the exposed group, there were inverse correlations between UPSIT® scores and treatment length (rho = −0.41; *p* = 0.01), an accumulated dose of ISO (rho = −0.39; *p* = 0.02), and NOSE scores (rho = −0.40; *p* = 0.02).

Regarding the substances tested in the UPSIT®, gasoline, coffee, orange, and wood were the ones with the highest identification errors among the ISO group, with less than 45% correct answers. Conversely, in the control group, motor oil, pickles, and grass were the substances with the most errors, achieving 60% correct answers (Table 2).

**Table 2:** Percentage of correct answers for each odorant (UPSIT®) between ISO and control groups.

Odorants	ISO (%) (n = 35)	Control (%) (n = 35)	*p*-value
01. Pizza	45.7	74.3	0.03
02. Bubble gum	94.3	97.1	>0.99
03. Menthol	91.4	94.3	>0.99
04. Cherry	97.1	94.3	>0.99
05. Motor oil	77.1	37.1	<0.01
06. Mint	100.0	97.1	>0.99
07. Banana	80.0	88.6	0.51
08. Clove	94.3	100.0	0.49
09. Leather	94.3	97.1	>0.99
10. Coconut	91.4	97.1	0.61
11. Onion	100.0	100.0	>0.99
12. Fruit juice	88.6	97.1	0.36
13. Baby powder	94.3	100.0	0.49
14. Jasmine	60.0	85.7	0.03
15. Cinnamon	100.0	97.1	>0.99
16. Gasoline	42.9	94.3	<0.01
17. Strawberry	82.9	94.3	0.26
18. Coffee	45.7	88.6	<0.01
19. Gingerbread	71.4	85.7	0.24
20. Apple	77.1	68.6	0.59
21. Perfume	97.1	100.0	>0.99
22. Flower	88.6	97.1	0.36
23. Peach	74.3	85.7	0.37
24. Tire rubber	100.0	91.4	0.24
25. Pickles	57.1	60.0	>0.99
26. Pineapple	97.1	100.0	>0.99
27. Raspberry	91.4	85.7	0.71
28. Orange	42.9	100.0	<0.01
29. Walnut	71.4	88.6	0.13
30. Watermelon	85.7	94.3	0.43
31. Solvent	88.6	77.1	0.34
32. Grass	65.7	60.0	0.80
33. Smoke	97.1	97.1	>0.99
34. Wood	42.9	82.9	<0.01
35. Grape	82.9	91.4	0.48
36. Garlic	97.1	97.1	>0.99
37. Soap	80.0	91.4	0.31
38. Natural gas	97.1	100.0	>0.99
39. Rose	77.1	85.7	0.54
40. Peanut	100.0	94.3	0.49

* Statistical significance accessed with Fisher exact test.

## Discussion

This study depicted an elevated prevalence of olfactory impairment among individuals exposed to ISO, despite a lack of self-reported olfactory complaints. The severity of the olfactory dysfunction was also correlated with the cumulative dose, length of treatment, and nasal obstructive symptoms, reinforcing the causality of the findings.

A Turkey investigation with 33 patients undergoing treatment with ISO assessed the olfactory function utilizing the Sniffin' Sticks Test (SST) before and after three months of treatment. They concluded that ISO improved the performance of patients in the scores of the olfactory test, despite the changes in the prevalence of hyposmia resulted in marginal significance. Nevertheless, the study was not controlled, and the SST has only 12 ranks which may not adequately define the extent of olfactory dysfunction, potentially skewing the perception of olfactory capability.^10^

Another olfactory test validated in Brazil (country of the present study) would be the Connecticut Chemosensory Clinical Research Center (CCCRC), but it was not carried out due to the authors' choice to use only the UPSIT®, which already entailed a significant time commitment for the research.^20^

The mechanism behind the alteration of the olfactory function by retinoids is not well known. Despite epithelial alterations leading to a dry nose, ISO activates Retinoic Acid Receptors (RARs) which are present in most nucleated cells, and the signaling through RARs regulates olfactory neural stem cells throughout life.^21^, ^22^ Some of these RA-activated cells are believed to be basal cells, located directly adjacent to the olfactory epithelium basement membrane, a position typically occupied by horizontal basal cells, which are presumed to be adult OE neural stem cells.^23^ In addition, the role of systemic inflammation induced by acne in olfactory performance should be elucidated.

The negative impacts of ISO on olfactory function could also be attributed to nasal obstruction, and other mucosal changes, as documented in related investigations regarding qualitative olfactory dysfunction, obstruction, epistaxis, and xerosis of the nasal mucosa, which might contribute to a decrease in the sense of smell.^9^, ^24^, ^25^ Indeed, an ideal balance of the nasal fluid is essential for good olfactory function. To improve the capture of odorants, a water-soluble medium would be more suitable, but not so exaggerated to avoid excessive congestion that might impede the odorants’ access to the olfactory receptors.^24^ Quantitative research into the dynamics of nasal fluid production and nasal obstruction before, during, and after ISO treatment could shed light on the impact of this therapy on these aspects and the associated compromise in olfactory function.

Interestingly, no correlation was observed between UPSIT® scores and socioeconomic factors such as family income or educational level in this sample, despite previous studies suggesting higher socioeconomic status may correlate with better olfactory function, probably due to their ability to use cognitive strategies and draw upon life experiences to differentiate the sense of smell.^26^ This discrepancy reassures a dominant impact of ISO on olfactory function over socioeconomic influences. Moreover, the expected decrease in smell with age was not detected in this study, nevertheless, our sample could be too young to perceive this effect.^27^

Other factors could influence the study such as smoking, respiratory infections and rhinitis.^14^ There were no smoking patients and only individuals more than 4 months after COVID infection without flu-like symptoms and without olfactory complaints entered the study. Patients with rhinitis were not in crisis and had no olfactory complaints either.

The impairment of olfactory function can also significantly affect quality of life and pose safety risks. This is particularly concerning for ISO users, who exhibited considerable difficulties in identifying critical odors like gasoline, highlighting the importance of these findings.^28^ These patients were advised to consult an otorhinolaryngologist for olfactory training, but the authors believe that the sense of smell will recover within 2 months after discontinuing ISO, similar to other side effects.

Despite the worldwide use of ISO for acne and other conditions,^29^ studies on its effects on olfactory function remain scarce. This study has limitations as it is monocentric and has a cross-sectional design. The nasal endoscopy examination was not performed, although the patients did not present clinical signs and symptoms of mouth breathing, snoring, or nasal deformity. However, this could be considered another limitation, as the procedure might have excluded other causes of olfactory dysfunction such as the presence of polyps and hypertrophy of the inferior turbinate.^30^ Longitudinal follow-up of patients with olfactory impairment after discontinuation of ISO is warranted to further understand the dynamics of this condition.

In conclusion, this study revealed a notable prevalence of olfactory dysfunction among ISO users, despite it was not accompanied by self-recognition of the condition. These results highlight the association between ISO exposure and reduced olfactory function, underscoring the importance of raising awareness by the clinicians and conducting further research to address this adverse effect.

## Authors’ contributions

1 Have made substantial contributions to conception and design, or acquisition of data, or analysis and interpretation of data: Rogério Nabor Kondo, Hélio Amante Miot, Marco Aurélio Fornazieri.
2 Been involved in drafting the manuscript or revising it critically for important intellectual content: Rogério Nabor Kondo, Hélio Amante Miot, Marco Aurélio Fornazieri.
3 Given final approval of the version to be published. Each author should have participated sufficiently in the work to take public responsibility for appropriate portions of the content: Rogério Nabor Kondo, Hélio Amante Miot, Elouise Zwirtes Frare, Ellen Cristine Duarte Garcia, Abner Hiraku Yamakami, Marco Aurélio Fornazieri.
4 Agreed to be accountable for all aspects of the work in ensuring that questions related to the accuracy or integrity of any part of the work are appropriately investigated and resolved: Rogério Nabor Kondo, Hélio Amante Miot, Elouise Zwirtes Frare, Ellen Cristine Duarte Garcia, Abner Hiraku Yamakami, Marco Aurélio Fornazieri.

## Institutional ethical approval

The protocol was approved by the Research Ethics Committee of the State University of Londrina (CAAE: #48734521.5.0000.5231).

## Data availability statement

The raw data of this study is available under contact with the corresponding author.

## Conflicts of interest

The authors declare no conflicts of interest.
